# The Astronomical Pulse of Global Extinction Events

**DOI:** 10.1100/tsw.2006.156

**Published:** 2006-06-15

**Authors:** David F.V. Lewis, Jean-Lou C.M. Dorne

**Affiliations:** ^1^ School of Biomedical and Molecular Sciences, University of Surrey, Guildford, Surrey, GU2 7XH, UK; ^2^ Clinical Pharmacology Group, Division of Developmental Origins of Health and Disease, Institute of Human Nutrition, School of Medicine, University of Southampton, Bassett Crescent East, Southampton, Hampshire, SO16 7PX, UK

**Keywords:** Mass Extinctions, Astronomical Cycles

## Abstract

The linkage between astronomical cycles and the periodicity of mass extinctions is reviewed and discussed. In particular, the apparent 26 million year cycle of global extinctions may be related to the motion of the solar system around the galaxy, especially perpendicular to the galactic plane. The potential relevance of Milankovitch cycles is also explored in the light of current evidence for the possible causes of extinction events over a geological timescale.

## INTRODUCTION

Interest in the apparent periodicity of mass species extinctions has been ongoing since the 1980s when Raup, Sepkoski and Boyajian[1,2,3,4,5,6] reported the likelihood of certain phased cycles of extinction events operating every 26 million years (my). These and other related studies, such as those involving crater impact analysis[7,8,9,10,11,12,13,14], were concerned with information covering the period from 250 million years ago (mya) to the present; however, the stratigraphic record is more reliable than earlier fossil data, which can sometimes be poor and incomplete.

The possibility of studying a somewhat longer time interval has, nevertheless, emerged from extensive Fourier analyses and other studies of extinctions that have occurred over the Phanerozoic aeon[7,8,9,10,11,12,13,14]. Such vast timescales indicate an astronomical origin and several regular cycles are currently known, including those originally proposed by Milankovitch to explain the existence of ice ages during recent geological time[15]. It is possible that oscillations in oceanic chemical balance correlate with glacial periods and interglacials as far back as 500 mya[16]. However, the fluctuations in the Earth's orbit, giving rise to Milankovitch cycles, are known to occur over much shorter timescales than the immense periods allotted to the geological record, which has been punctuated by mass extinctions[17,18,19,20,21,22,23,24,25,26].

It is common for entirely new species to emerge following global extinction events, which implies that environmental pressures bring about evolutionary changes[27]. For example, the so-called C-T event, which heralded the demise of the higher saurians 65 mya, could well have resulted in the mammalian radiation over recent geological time[28]. The once-regarded as overly speculative theory of bolide impacts has recently been resurrected in the light of the Shoemaker-Levy 9 cometary Jovian collisions in 1994 as representing the most likely cause of the extinction event 65 mya[20], with its associated iridium anomaly in the stratigraphic record[28].

It is possible that there are regular intervals of time when the number of bolide impacts are at a maximum due to the movement of the solar system within the galactic arm, both laterally and longitudinally. The likelihood of both types of motion may lead to different, but regular, periods of maximal bolide impacts within the solar system's planets and moons. If this is the case, then such times of high-impact incidence will probably have a deleterious effect on the terrestrial biosphere, thus leading to species extinctions at fairly regular intervals of time, albeit large.

Previous authors have hinted at astronomical causes[8,9,10,14,29] and it is possible that, from an analysis of several orbital fluctuations with respect to both the Earth's motion and that of the solar system as a whole, one may be able to associate such quantities with the observed extinction patterns. However, some of the extinction events are global in nature and, therefore, one should also consider additional causation theories to account for such higher-order periodicity, including sea level changes and temperature fluctuations that may be related to the supercontinental cycle, for example.

An analysis reported in the literature shows a 13(±2)-my cycle based on crater impact data and further consideration of such information indicates a 33(±3)-my component. This cycle length could coincide with the galactic axial oscillation frequency, together with a higher-order periodicity of 260 my, which is roughly in line with the galactic “year’ of 250(±50) my[10]. Pulses of such regularity point to terrestrial orbital changes that coincide every 13 or 26 my to produce climatic events, such as the ice ages and changes in sea level[30,31,32,33,34,35,36]. These fluctuations are likely to affect the natural optimum conditions benefited by certain species; however, these may well have already been in decline for a number of reasons, including evolutionary pressures[37].

Although it is known that there is some degree of discrepancy between the timescale for geological periods reported by different groups of workers[38,39], an error of ±4 my is quite acceptable when considering events of such immense age, especially those periods earlier than 250 mya where the fossil record is less clearly defined[38,39].

## Milankovitch Cycles

Glaciations and accompanying sea level changes over the last million years can be explained satisfactorily in terms of the so-called Milankovitch cycles[15]. These are, in fact, changes in the Earth's orbital characteristics brought about by the gravitational influence of other bodies in the solar system including the sun, moon, and possibly one or more of the large outer planets. Indeed, the regular pattern of sunspot cycles may be linked to Jupiter's orbital period of approximately 11 years, although conclusive evidence is scarce.

The regularity of ice ages over the last million years concords with sea level changes and also points to average terrestrial temperature variations which, presumably, have been caused by a periodicity in the solar output reaching the Earth. Apparently, these cyclical variations occur with periodicities closely related to the three examples of terrestrial orbit fluctuations originally proposed by Milankovitch[15]. Interestingly, many of the current explanations of mass extinction events relate to global temperature variations, usually involving a decrease.

These three cycles appear to coincide every 26 my with a peak-trough period of 13 my. This peak represents the smallest possible timescale for constructive reinforcement (analogous to the interference pattern of wave motion) of the three main cycles of axial tilt, precession, and eccentricity pulsing at 41,000, 23,000, and 95,000 years[40], respectively. These cycles would be expected to be in phase every 26 my, according to a simple factor analysis. Inspection of the extinction pattern reveals a recurrence of the 26-my feature reported by Raup, Sepkoski and Boyajian[1,2,3,4,5,6] and, occasionally, a 13-my period of extinction events can be observed even though it does not necessarily represent *global* mass extinctions (see

Table 1 and Fig. 1, which correlate the data presented). The magnitude of the extinction event appears to be dependent on additional cyclical factors such as the galactic tilt frequency of 33 my and the higher-order pulse of the solar system's rotation within the galactic plane (~220 my). These are known to affect the integrity of the Oort Cloud of comets that lies just outside the solar system[41]. Disturbance of the Kuiper belt asteroids by gravitational aberrations due to cyclic variations such as the solar system's passage through the galactic spiral arms (or the galactic axial tilt) will also give rise to an increase in bolide impacts[41], which could thus cause biological extinctions with a phased regularity. The Oort Could tends to be the source of new cometary material, whereas the somewhat closer Kuiper belt comprises a reservoir for most periodic comets, although other Kuiper belt objects (KBOs) are also known[41].

**TABLE 1**
**Catalogue of Extinctions During the Phanerozoic Aeon[1,2]**

| Event Time (mya) | Estimate (mya) | 13-my Multiple Period |
|---|---|---|
| 11.3 | 13 | 1 |
| 38 | 39 | 3 |
| 65 | 65 | 5 |
| 91 | 91 | 7 |
| 114 | 117 | 9 |
| 144 | 143 | 11 |
| 170 | 169 | 13 |
| 194 | 195 | 15 |
| 208 | 208 | 16 |
| 219/225 | 221 | 17 |
| 248 | 247 | 19 |
| 289 | 286 | 22 |
| ~300 | 299 | 23 |
| ~330 | 325 | 25 |
| 360–368 | 364 | 28 |
| 408 | 403 | 31 |
| 438–440 | 442 | 34 |
| 505 | 507 | 39 |
| 570 | 572 | 44 |
| 630 | 637 | 49 |

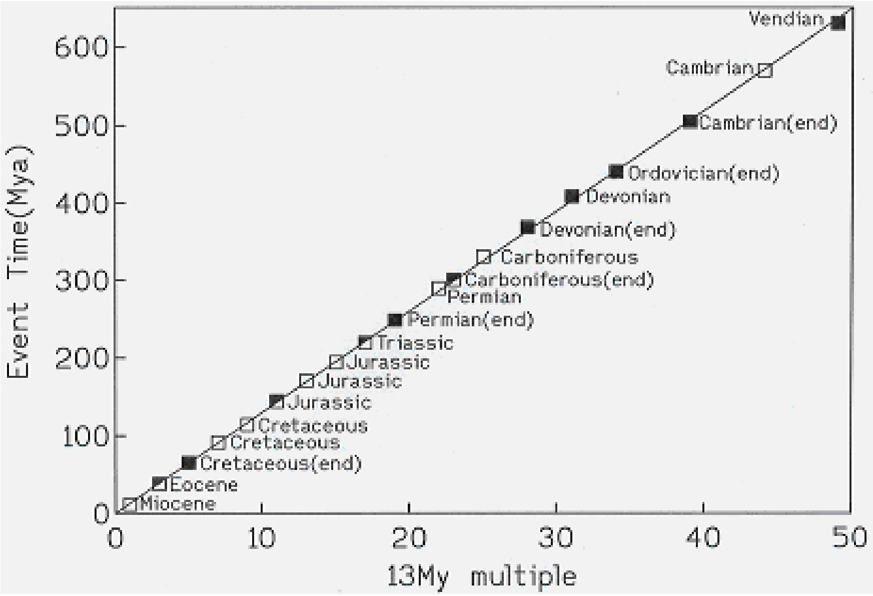

**FIGURE 1.** A linear plot of extinction event time (mya) vs. multiple of 13 my for mass extinctions over the Phanerozoic aeon. Major extinction events are shown in black whereas moderate ones are in black and white. The data used are from Table 1.

## Galactic Cycles

The sun's immediate neighbourhood, with a galactic position of about 8200 parsecs from the centre (1 parsec = 3.2616 light years or $3.0857 \times 10^{16}$m) and currently lying at a few parsecs north of the galactic plane, place it in one of the huge spiral arms of the Milky Way, specifically the Cygnus-Orion arm[42]. The solar system revolves about the galactic centre at a velocity of ~250 $kms^{-1}$. A simple calculation shows that the galactic "year", or one complete revolution of the solar system around the galactic centre, is about 201.5 my based on current astronomical data; however, somewhat longer periods of 220, 225, and 240 my have also been reported[43].

The 13- or 26-my periodicity is fairly satisfactory to describe the overall pattern, but does not provide an explanation for the major peaks in the extinction record. These may, however, arise from constructive interference of several cycles of higher order, such as the galactic axial tilt (galactic "wobble") and the ~225-my period for the solar system's revolution of the galaxy described previously. Interestingly, some of the gaps between major extinction events are between 30 and 32 my, whereas a major difference of ~220 my is apparent for the extinctions 440–219, 368–144, and 289–65 mya. Other slightly shorter time intervals of ~200 my occur for the extinction events 630–438, 440–248, 248–39, and 219–11 mya, although the galactic revolution of the solar system may not have been constant over geological time. The effect of the solar system's galactic rotation will probably have been experienced by the developing Earth and its biota over the past 4.75 billion years, such that key events in the life of the planet, i.e., species evolution, could be synchronistically linked to this and other cycles. The timings and duration of geological ages have themselves largely been determined on the basis of species extinctions and, consequently, there is apparently some degree of 13-my periodicity in their pattern. Table 2 shows that the beginning of each geologic period is associated with a multiple of 13 my, whereas Table 3 indicates those occasions where mass extinctions have been associated with loss in both species and genera. 'Table 4 indicates where iridium anomalies have been identified for six mass extinction events, together with a summary of possible causes for the extinctions, which point to global cooling in general. A plot of the data shown in Table 3 is presented in Fig. 2 and indicates the complex nature of these overlapping cycles.

For example, there may be a linkage between the rise in atmospheric oxygen levels and the galactic rotational period, and these are presented in Table 5, together with other evolutionary events correlated with the 225-my pulse.

**TABLE 2**
**Phanerozoic Aeon and its Periods[37]**

| Period | mya | 13-my Multiple |
|---|---|---|
| Quaternary | 1.8 | 0 |
| Tertiary | 65 | 5 |
| Cretaceous | 144 | 11 |
| Jurassic | 206 | 16 |
| Triassic | 251 | 19 |
| Permian | 290 | 22 |
| Carboniferous | 353.7 | 27 |
| Devonian | 408.5 | 31 |
| Silurian | 439 | 34 |
| Ordovician | 495 | 39 |
| Cambrian | 543 | 42 |

**TABLE 3**
**Mass Extinctions During the Phanerozoic Aeon[37,40,52,53,59,60]**

| Event | mya | 13-my Multiple | % Species[a] | % Genera[a] | % Families[a] | % Families[b] |
|---|---|---|---|---|---|---|
| Late Eocene | 35.4 | 3 | 35 (±8) | 15 | — | — |
| End Cretaceous | 65.0 | 5 | 76 (±5) | 47 (±4.1) | 16 (±1.5) | 15*, 17† |
| Late Cretaceous | 90.4 | 7 | 53 (±7) | 26 | — | — |
| End Jurassic | 145.6 | 11 | 45 (±7.5) | 21 | — | — |
| Early Jurassic | 187.0 | 14 | 53 (±7) | 26 | — | — |
| End Triassic | 208.0 | 16 | 80 (±4) | 53 (±4.4) | 22 (±1.7) | 20*, 23† |
| End Permian | 245.0 | 19 | 95 (±2) | 82 (±3.8) | 51 (±2.3) | 50*, 57† |
| Late Devonian | 367.0 | 28 | 83 (±4) | 57 (±3.3) | 22 (±1.7) | 21*, 19† |
| End Ordovician | 439.0 | 34 | 85 (±3) | 60 (±4.4) | 26 (±1.9) | 22*, 27† |

a Binomial standard errors in the estimates are shown in parentheses[37,53,60], although the errors for some of the genera data were not available for the less-dramatic extinction events.

b Comparable values for the percentage extinction of Families are taken from Doyle et al.[40](*) and Sepkoski[59](†) although standard errors were unavailable in these cases.

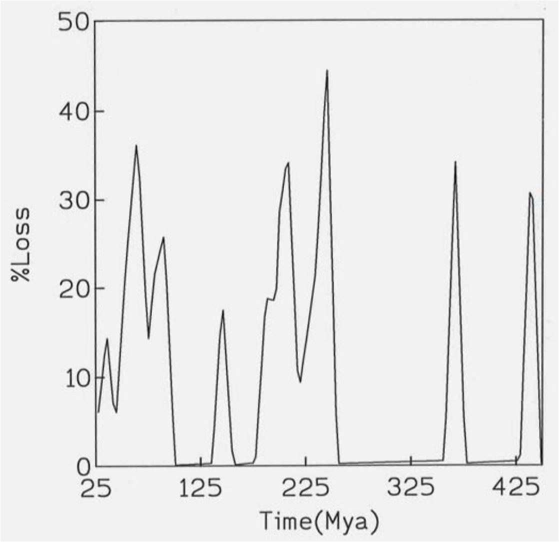

**FIGURE 2.** The percentage loss of species and genera for a number of major mass extinctions over the last 440 my vs. event time (mya) are shown as a peak-and-trough diagram. The data used are from Table 3.

**TABLE 4**
**Mass Extinctions and Correlations with Iridium Levels and Climate Change[28,40]**

| Boundary | mya | Iridium Anomaly | Likely Causes |
|---|---|---|---|
| Eocene-Oligocene | 38 | Small spike | Cooling, glaciation |
| Cretaceous-Tertiary | 65 | Spike | Bolide impact |
| Permo-Triassic | 245 | Spike | Refrigeration, regression |
| Late Devonian | 367 | Small spike | Global cooling |
| Ordovician-Silurian | 439 | Normal | Growth of Gondwanan Ice Sheet |
| Precambrian-Cambrian | 570 | Small spike | Global cooling |

We have explored extinctions of the Phanerozoic aeon and up to (and including) 630 mya, which corresponds to the start of the Vendian era. Table 1 and Fig. 1 illustrate these events with the relevant information compiled from a number of sources[28,38,40], together with those multiples of 13 my coinciding within ±4 my of the actual extinction event and their correlation. The supercontinental cycle of plate tectonic movement has been shown to bring about a regular opening and closing of the continents, occurring with a periodicity of about 440 my[40]. Continental drift is also thought to have given rise to ordinal diversification[44,45] of metazoans during the Phanerozoic aeon and this mechanism may also explain certain species extinctions. Interestingly, this cycle is about twice the length of the galactic "year" and one might hypothesize that, like the 26- and 13-my patterns, the peak-trough relationship corresponds to a regular cycle of "wavelength" 440 my. The process of evolution and those of specific biological systems, such as the cytochromes P450[46,47,48], is complex, although it is possible that markers such as glacial cycles, sea level changes, and the oxygen isotopic ratios[49] can be employed, together with changes in microbiota[50,51], to substantiate the potential causes of extinction events and their possible linkage to orbital cycles. Over the last 20 years or more, various authors have reported on extinction events[32,33,34,35,36,37,38,39,40,41,42,43,44,45,46,47,48,49,50,51,52,53,54,55,56,57,58,59,60] and the case for a periodicity in global mass extinctions is strong, especially from the work of Sepkoski and colleagues[1,2,3,4,5,6,39,56,57,58,59,60]. Milankovitch[15] has shown that the ice ages can be analysed satisfactorily from a detailed consideration of terrestrial orbital cycles and, therefore, one may imagine that these and other astronomical variations, including the solar system's galactic orbit, could help to explain the observed extinctions of species over geological time.

**TABLE 5**
**Timeline of Earth History[28,38,40]**

| mya | Geological and Evolutionary Events | 225-my Multiple |
|---|---|---|
| 4750 | Formation of the Earth | 21 |
| 4525 | Earth-Moon System formed via large body impact, crustal formation | 20 |
| 4300 | Ocean formation | 19 |
| 4075 | Possible formation of life | 18 |
| 3850 | Bolide Impacts | 17 |
| 3625 | Life established | 16 |
| 3400 | Stromatolites develop | 15 |
| 3175 | Blue-green algae develop | 14 |
| 2950 | Orogeny | 13 |
| 2725 | Glacial episode, onset of plate tectonics | 12 |
| 2500 | Orogeny, oxygen present in atmosphere | 11 |
| 2275 | Glacial episode, supercontinent forms | 10 |
| 2050 | Eubacteria-eukaryote divergence, protozoa develop | 9 |
| 1825 | Archaebacteria-eukaryote divergence (extinctions, banded ironstones) | 8 |
| 1600 | Start of Mesoproterozoic, photosynthesis triggers rise in atmospheric oxygen | 7 |
| 1375 | Megascopic algae develop | 6 |
| 1150 | Nonesuch shale deposits, plant-animal divergence | 5 |
| 925 | Fungi-animal divergence, microbiota develop | 4 |
| 700 | Glacial episode, extinctions | 3 |
| 475 | Extinctions, ammonites and invertebrates develop | 2 |
| 250 | Major global extinctions, orogeny | 1 |
| 25 | Extinctions, orogeny | N/A |

*Note:* The period of solar system's rotation about the galaxy is about 225 my and there would, therefore, have been 21 galactic rotations since the Earth was formed.

## CONCLUSIONS

The solar system's revolution about the galactic centre may not have remained constant over the last billion years and additional factors, such as the rise in atmospheric oxygen and fluctuations in solar output, should be considered to provide an explanation of extinction patterns. However, the essentially gravitational sources of regular variation appear to be primarily responsible for mass extinctions either by changing the solar radiation incidence on Earth (Milankovitch effect) or by initiating bolide impact peaks via galactic cycles, as described previously. In conclusion, far from being a taxonomic artifact[29], it is likely that periodic extinction of the biota[52,53,54] arises from the pulse in astronomical cycles.

## ACKNOWLEDGEMENTS

The financial support of ExxonMobil Biomedical Sciences, Inc. and the British Technology Group is acknowledged by one of us (DFVL).
